# Publisher Correction: Regulation of cellular sterol homeostasis by the oxygen responsive noncoding RNA *lincNORS*

**DOI:** 10.1038/s41467-020-19708-7

**Published:** 2020-11-06

**Authors:** Xue Wu, Cristina M. Niculite, Mihai Bogdan Preda, Annalisa Rossi, Toma Tebaldi, Elena Butoi, Mattie K. White, Oana M. Tudoran, Daniela N. Petrusca, Amber S. Jannasch, William P. Bone, Xingyue Zong, Fang Fang, Alexandrina Burlacu, Michelle T. Paulsen, Brad A. Hancock, George E. Sandusky, Sumegha Mitra, Melissa L. Fishel, Aaron Buechlein, Cristina Ivan, Spyros Oikonomopoulos, Myriam Gorospe, Amber Mosley, Milan Radovich, Utpal P. Davé, Jiannis Ragoussis, Kenneth P. Nephew, Bernard Mari, Alan McIntyre, Heiko Konig, Mats Ljungman, Diana L. Cousminer, Paolo Macchi, Mircea Ivan

**Affiliations:** 1 Department of Microbiology and Immunology, Indiana University School of Medicine, Indianapolis, IN 46202 USA; 2 Department of Medicine, Indiana University School of Medicine, Indianapolis, IN 46202 USA; 3 “Victor Babes” National Institute of Pathology, Bucharest, Romania; 4 Institute of Cellular Biology and Pathology “Nicolae Simionescu”, Bucharest, Romania; 5 Laboratory of Molecular and Cellular Neurobiology, Department of Cellular, Computational and Integrative Biology—CIBIO, University of Trento, Trento, Italy; 6 Laboratory of Translational Genomics, Department of Cellular, Computational and Integrative Biology—CIBIO, University of Trento, Trento, Italy; 7 Yale Cancer Center, Yale University School of Medicine, New Haven, CT 06520 USA; 8 The Oncology Institute “Prof Dr. Ion Chiricuta”, Cluj-Napoca, Romania; 9 Metabolite Profiling Facility, Bindley Bioscience Center, Purdue University, West Lafayette, IN 47907 USA; 10 Department of Genetics, Department of Systems Pharmacology and Translational Therapeutics, Institute of Translational Medicine and Therapeutics, Perelman School of Medicine, University of Pennsylvania, Philadelphia, PA 19104 USA; 11 Department of Cellular and Integrative Physiology, Indiana University School of Medicine, Indianapolis, IN USA; 12 Departments of Radiation Oncology and Environmental Health Sciences, Center for RNA Biomedicine, University of Michigan, Ann Arbor, MI 48109 USA; 13 Department of Surgery, Indiana University School of Medicine, Indianapolis, IN 46202 USA; 14 Department of Pathology and Laboratory Medicine, Indiana University School of Medicine, Indianapolis, IN 46202 USA; 15 Melvin and Bren Simon Cancer Center, Indiana University, Indianapolis, IN USA; 16 Department of Obstetrics and Gynecology, Indiana University School of Medicine, Indianapolis, IN 46202 USA; 17 Department of Pharmacology and Toxicology, Department of Pediatrics, Wells Center for Pediatric Research, Indiana University School of Medicine, Indianapolis, IN 46202 USA; 18 Indiana University Center for Genomics and Bioinformatics, Bloomington, IN 47405 USA; 19 Center for RNA Interference and Non-coding RNAs, The University of Texas MD Anderson Cancer Center, Houston, TX 77030 USA; 20 Department of Human Genetics, McGill University and Genome Quebec Innovation Centre, McGill University, Montréal, QC Canada; 21 Laboratory of Genetics and Genomics, National Institute on Aging, National Institutes of Health, Baltimore, MD 21224 USA; 22 Department of Biochemistry and Molecular Biology, Indiana University School of Medicine, Indianapolis, IN 46202 USA; 23 Medical Sciences, Indiana University School of Medicine, Bloomington, IN USA; 24 CNRS, IPMC, FHU-OncoAge, Université Côte d’Azur, Valbonne, France; 25 Rogel Cancer Center, University of Michigan, Ann Arbor, MI 48109 USA; 26 Centre for Cancer Sciences, Biodiscovery Institute, Nottingham University, Nottingham, UK; 27 Division of Human Genetics, Department of Pediatrics, The Children’s Hospital of Philadelphia, Perelman School of Medicine, University of Pennsylvania, Philadelphia, PA 19104 USA

**Keywords:** Long non-coding RNAs, Homeostasis

Correction to: *Nature Communications* 10.1038/s41467-020-18411-x, published online 21 September 2020.

The original version of this Article contained an error in Fig. 7b, where the *p* value for the association of the rs246185 SNP with MKL2 expression was accidentally copied during typesetting from the above panel, showing association with lincNORS, and incorrectly reported as 7.64e − 7. The correct *p* value is 1.45e − 1.

This has now been corrected in both the PDF and HTML versions of the Article.

