# Supplementary material for: Regulation of cellular sterol homeostasis by the oxygen responsive noncoding RNA lincNORS
Source: Nat Commun. 2020 Sep 21;11:4755. doi: 10.1038/s41467-020-18411-x (PMC7505984; doi:10.1038/s41467-020-18411-x)
Supplement: Supplementary file 1 — Supplementary Information [file 41467_2020_18411_MOESM1_ESM.pdf]

Supplementary Information

Regulation of cellular sterol homeostasis by the oxygen responsive noncoding RNA *lincNORS*

Wu et al.

This PDF file includes Supplementary Figures 1 to 13

Supplementary Figure 1

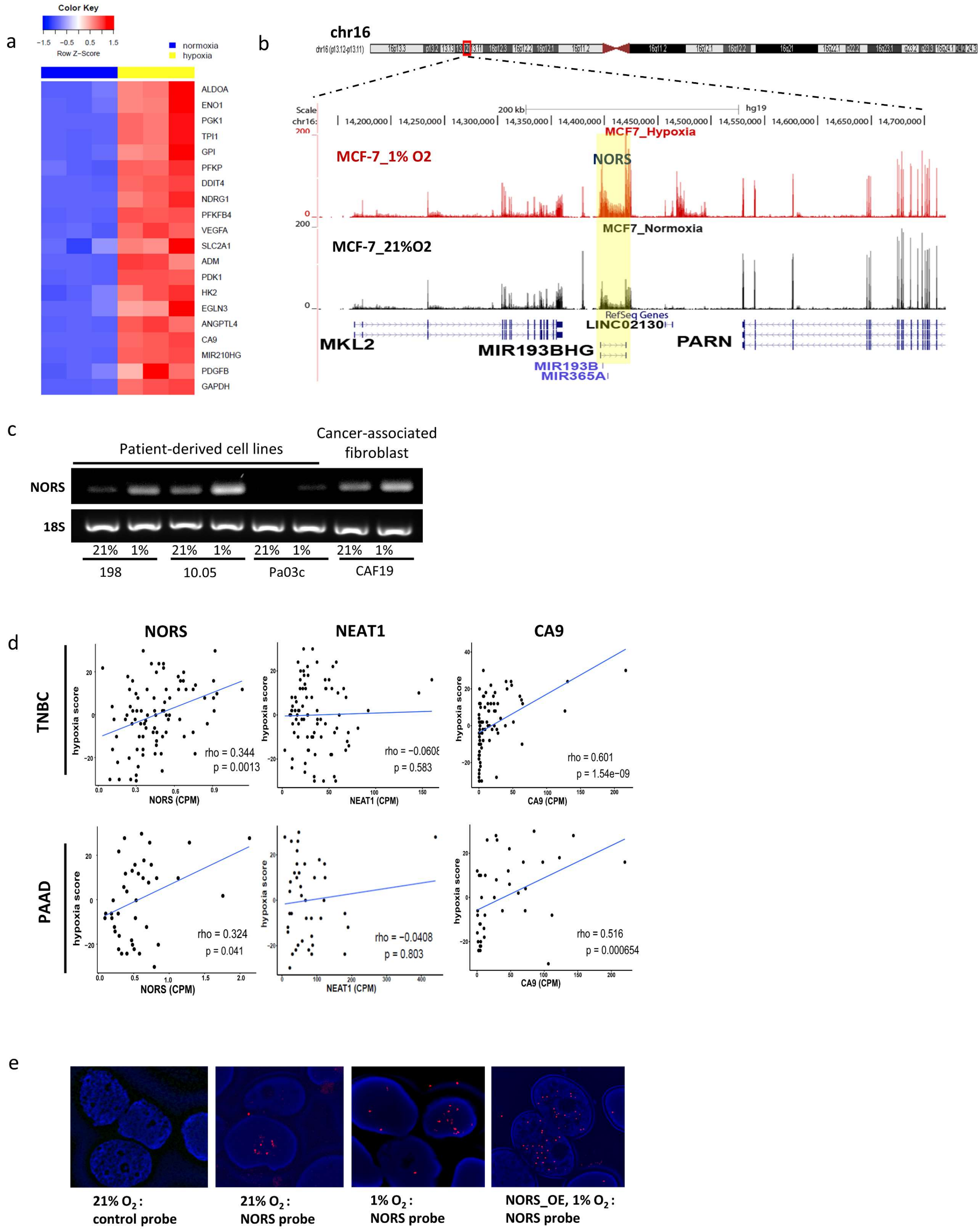

Supplementary Figure 1. ***lincNORS*** **is an oxygen-regulated, nucleus-located lncRNA.**

(a) Heatmap showing the expression of a panel of hypoxia signature genes in MCF-7 cells under hypoxia (1% O_2, 24 hrs) versus normoxia (21% O_2, 24 hrs). Color intensity represents RPKM scaled by the row. Source data are provided as a Source Data file.

(b) UCSC genome browser view of total RNA at the *lincNORS(MIR193BHG)* locus in MCF-7 cells under 21% O_2 (black) and 1% O_2 (red) detected by RNA-seq. *lincNORS/MIR193BHG* is highlighted in yellow.

(c) Validation of *lincNORS* induction by hypoxia in patient-derived pancreatic ductal adenocarcinoma cell lines and cancer associated fibroblast. Cells were cultured in 21% or 0.2% O_2 for 24 hours and *lincNORS* RNA level was measured by RT-PCR using the same cycle number across all samples. A representative image from three independent experiments was shown.

(d) Expression of *lincNORS, NEAT1 or CA9* as a function of the tumor hypoxia score in 84 TNBC and 41 PAAD tumor samples from TCGA. Spearman's correlation and p-value were reported.

(e) RNAscope detection of *lincNORS* RNA in MCF-7 cells and MCF-7 with stable overexpression of *lincNORS* (*lincNORS*: red; DAPI: blue). Representative images from three independent experiments were shown.

Supplementary Figure 2

a

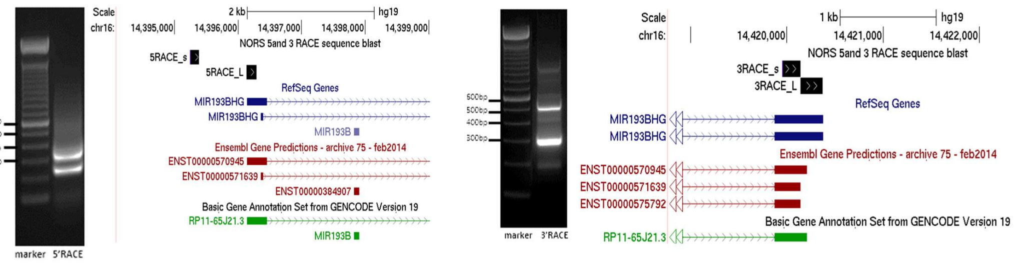

b

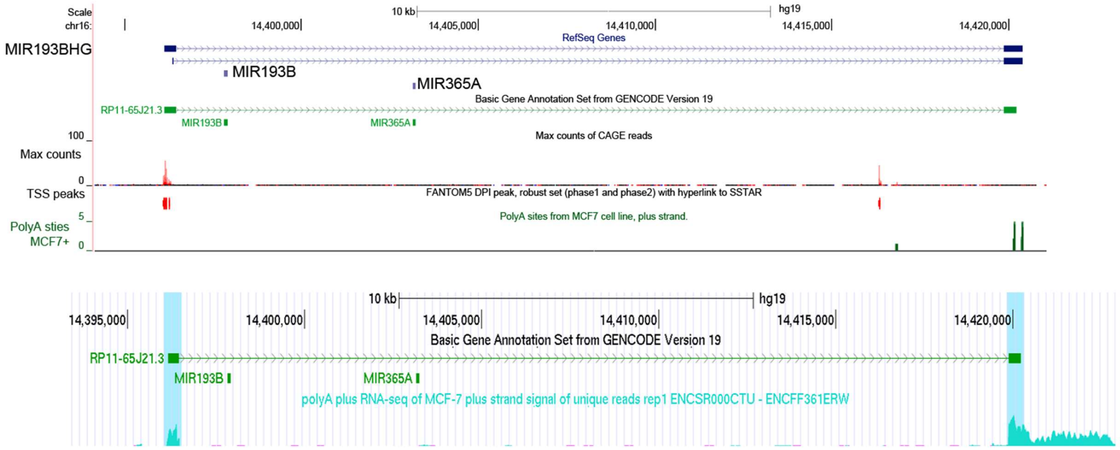

c

Metric	Raw result	Interpretation
PRIDE reprocessing 2.0	0	non-coding
Lee translation initiation sites	0	non-coding
PhyloCSF score	-193.6729	non-coding
CPAT coding probability	1.88%	non-coding
Bazzini small ORFs	0	non-coding

Supplementary Figure 2. Characterization of *lincNORS* transcript.

(a) 5' RLM-RACE (left) and 3' RACE (right) were performed to determine the start and end sites of *lincNORS* transcript. Representative agarose gel images from three independent experiments show 5' RACE and 3' RACE final PCR products as indicated. Major products from the PCR were sequenced and aligned against the human genome for comparison to existing annotations in the indicated databases.

(b) UCSC genome browser displaying *lincNORS(MIR193BHG)* transcription orientation and start/end sites. Tracks presented are: TSS peaks track from FANTOM5 project; poly(A) track from ENCODE Cancer genome polyA site & usage data; CSHL long RNA-seq.

(c) Coding potential of *lincNORS* was estimated using PRIDE reprocessing 2.0, Lee translation initiation sites, PhyloCSF score, Coding-Potential Assessment Tool (CPAT), and Bazzini small ORFs.

Supplementary Figure 3

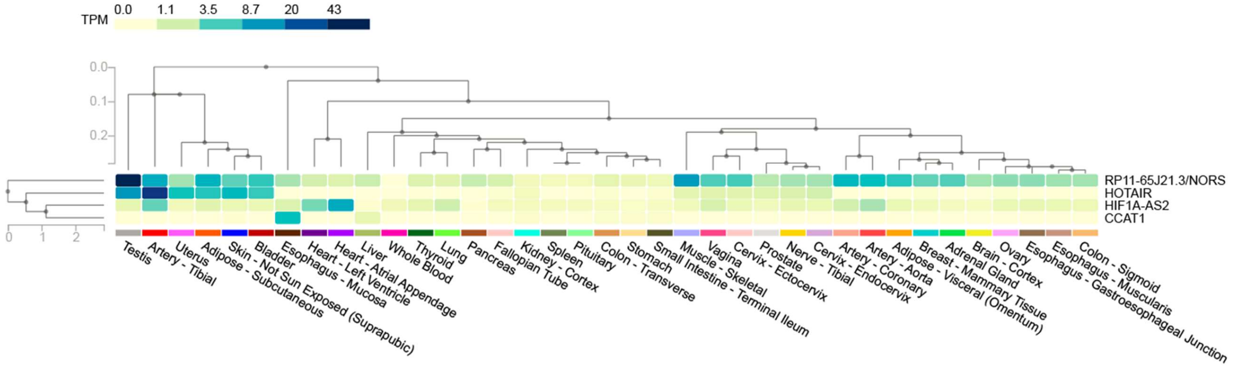

Supplementary Figure 3. Expression of *lincNORS* and several previously reported lncRNAs across normal tissues according to the GTEx database.

Supplementary Figure 4

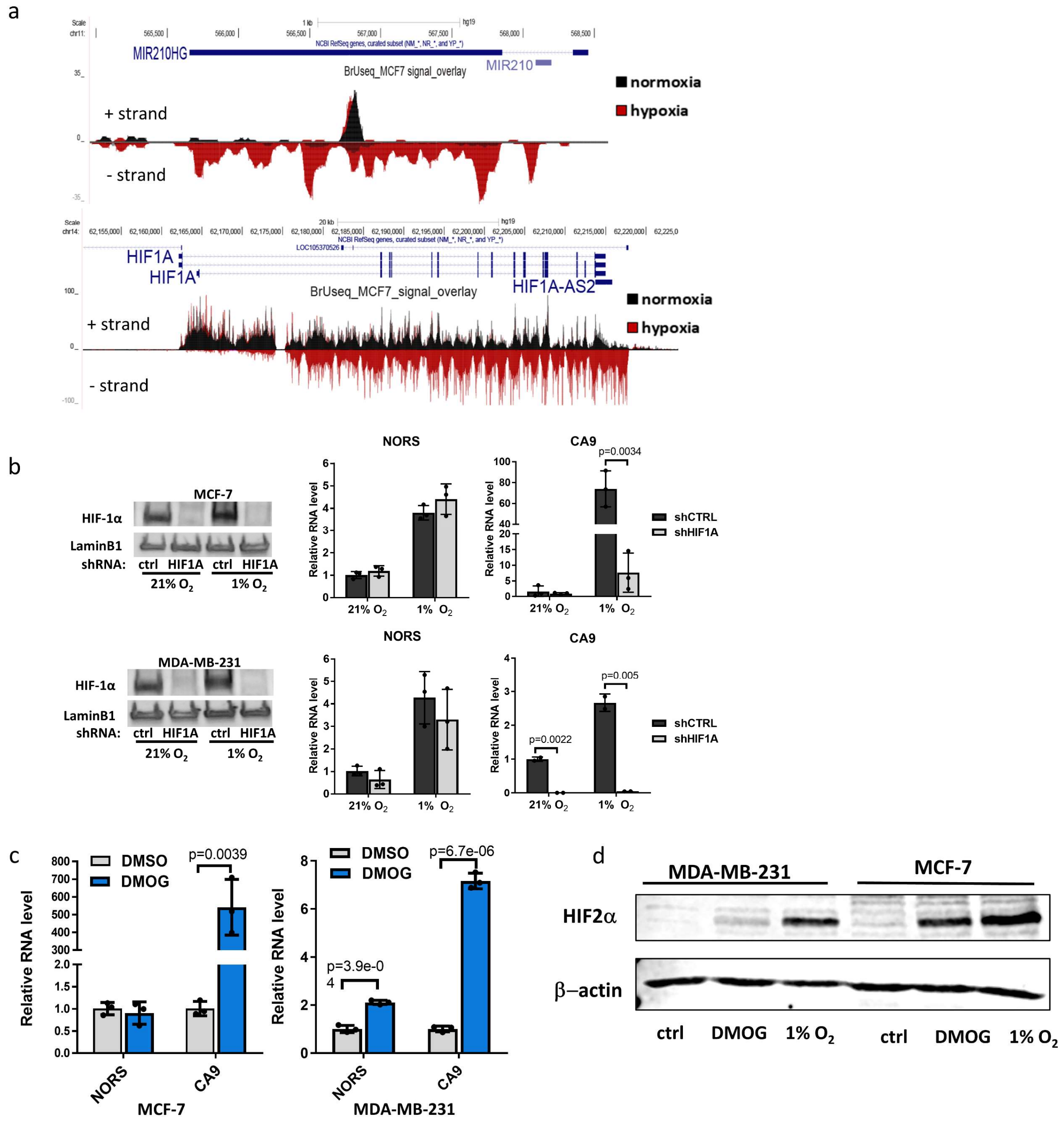

Supplementary Figure 4. *lincNORS* expression is regulated by HIF-2α.

(a) UCSC genome browser view of Bru-seq showing newly synthesized RNA at MIR210HG and HIF1A/HIF1A-AS2 loci.

(b) qPCR analysis of *lincNORS* expression in MCF-7 (top) and MDA-MB-231 (bottom) cells with stable HIF-1α knockdown. MCF-7 and MDA-MB-231 cells stably expressing HIF-1α shRNA or control shRNA were cultured in 21% O2 or 1% O2 for 48hrs. HIF-1α knockdown was confirmed

by western blot and representative results are shown. Data are shown as mean ± SD from three biological replicates (two-sided Student's t-test).

(c) qPCR analysis of *lincNORS* and CA9 expression in MCF-7 and MDA-MB-231 cells treated with 1mM DMOG or vehicle control DMSO for 24 hrs. Data are shown as mean ± SD from three biological replicates (two-sided Student's t-test).

(d) Western blot showing HIF-2α expression in MCF-7 and MDA-MB-231 cells treated with vehicle control (DMSO), 1mM DMOG or 1% O2 for 24 hrs. A representative image from three independent experiments was shown.

Supplementary Figure 5

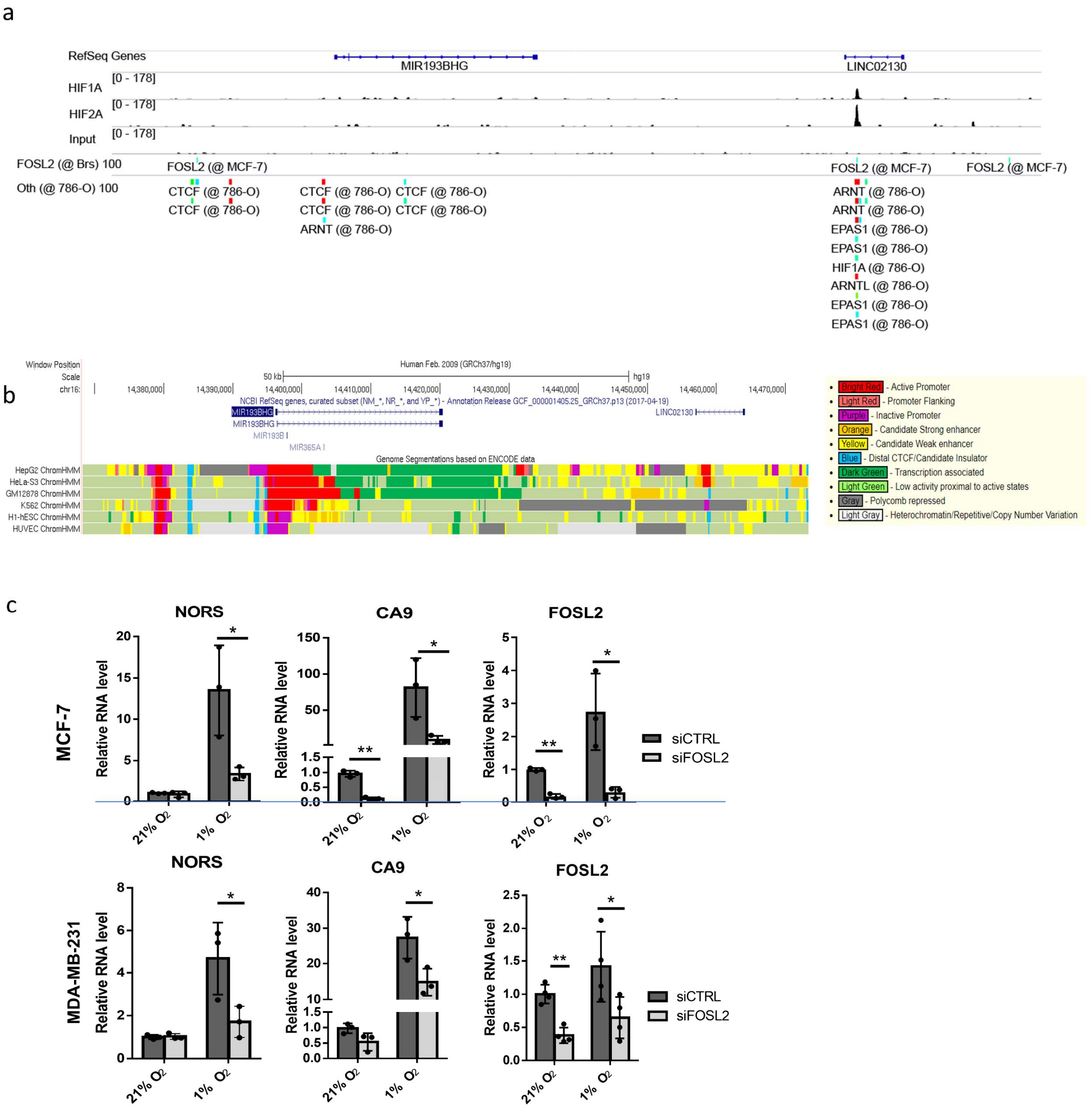

Supplementary Figure 5. HIF-1α and HIF-2α bind to a distant enhancer region LINC02130.

(a) IGV view of HIF-1α, HIF-2α (GSE28352), and FOSL2 (ChIP-Atlas) binding in MCF-7 cells and transcription factors binding in 786-O cells (ChIP-Atlas) at *lincNORS* and LINC02130 loci.

(b) UCSC genome browser view of genome segmentation from ENCODE. The candidate annotations and the associated segment colors are displayed on the right.

(c) qPCR analysis of *lincNORS* expression in MCF-7 and MDA-MB-231 cells with FOSL2 knockdown by siRNA. MCF-7 and MDA-MB-231 transfected with control or FOSL2 siRNA were cultured in 21% O2 or 1% O2 for 48hrs. FOSL2 knockdown was confirmed by qPCR. Data are shown as mean ± SD from three biological replicates (*p < 0.05, two-sided Student's t-test). Source data and exact P values are provided in the Source Data file.

Supplementary Figure 6

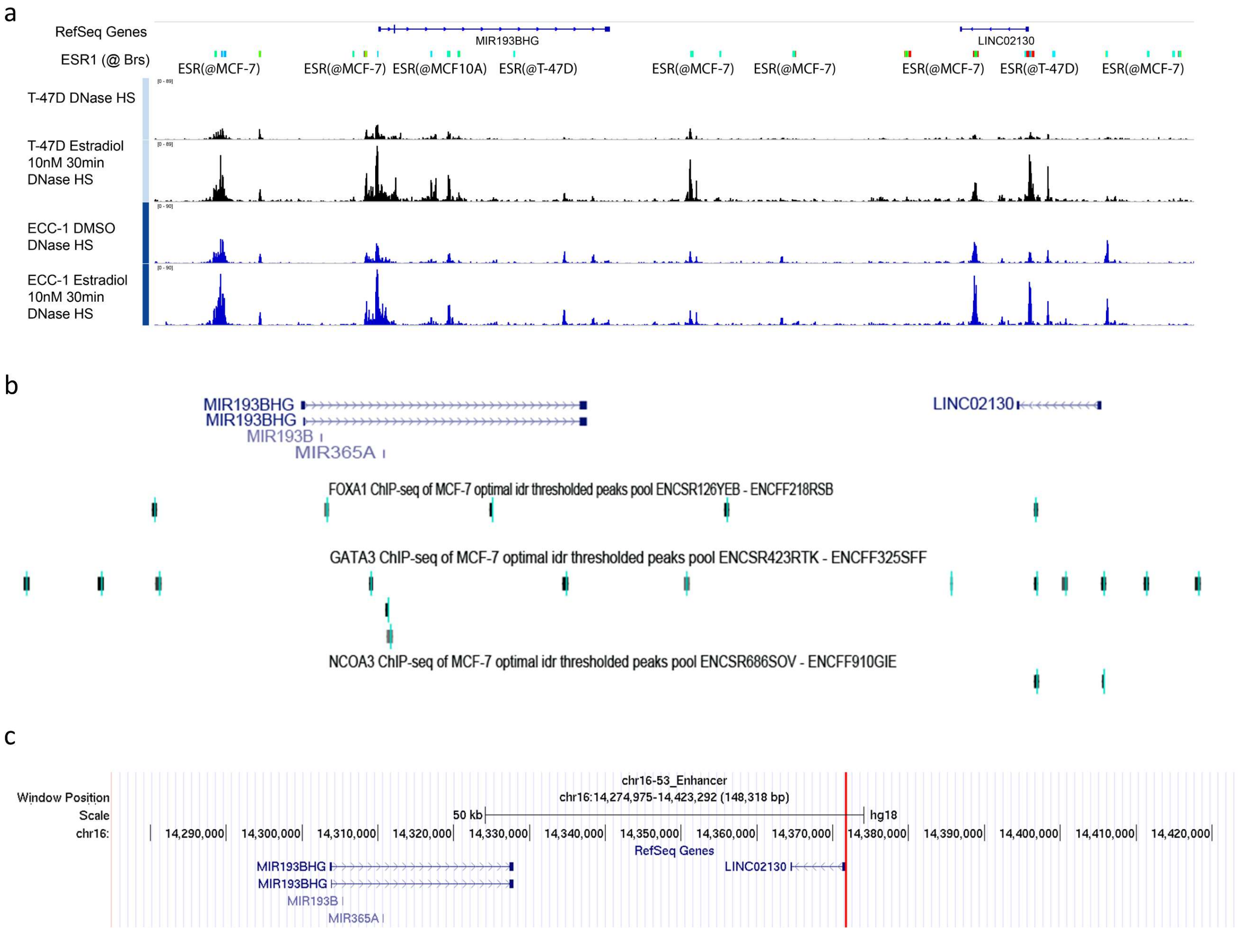

Supplementary Figure 6. ESR1 and ESR1 pioneer factors binding near the lincNORS locus.

(a) IGV view of ESR1 binding (ChIP-Atlas) and DNase I hypersensitivity (ENCODE) after E2 treatment around lincNORS locus.

(b) UCSC genome browser view of ESR1 pioneer factors and functional partners FOXA1, GATA3, and NCOA3/SRC3 binding in lincNORS-LINC02130 region from ENCODE.

(c) Mega trans ER enhancer near LINC02130 reported by Rosenfeld visualized on UCSC genome browser.

Supplementary Figure 7

a

CCGTCGGCTGCGCGCTCTCTGCAGAAAAGGATTTTCTACGCTCCCCGCCCATCACTTGGATCTCGTCT
GGGGAGGGGCGGATTTTTTTTCTGGAAGTACCCTTCTTCTCCACGGTGGAGGATTTAAGGCTGGGCT
CCAATCGAGGCGGAGGCTGCAGGGCCCGTGCACCCCCGGAGGCGGCAGCGGCGATAAACCTGCCAG
TAATTTCAGCAAATGAAACGTGGAACACGTCAAGGCCAGAGGATGGAAAAGGCTCCAGTTTTGATAA
AAGGAGGCCTGCTTGGCTTGGAATCCTCTCTCCTGCTGGTTGGGAGATGCAAAGATGTTTCCAGAGA
GGGGCTGATGAATTGAGGGGAAAGAAATGAGCCCAGTATGAGTCCCCTTTCAGGGCTGAGCGTGTA
TAAAACCAAACAACTTGGAACCGCTCCAAGAGAGGGGATTAAAGCAACATGTTATTCTGAGTGATTG
CTTAATTTATTGAGCTGCGGCTGGATCTGTAATGAAATACAGCCCTTGTAACTGATAACCTCCTGCTGC
CATTGAACTTCTACAATTAAGGAATATTTTCTGAGTTTCTCTGGAACGGCTCTGAATTTTTAGCCTCTG
TGGTAGGGTGCTTCTGAACATTTGTTTTCCAGGCAATTTTTTTTGAGTATTAGGCTGATGTTAATAAAT
AAGCAGCATTTTATTGAGT

siNORS_s
siNORS_w
shNORS

b

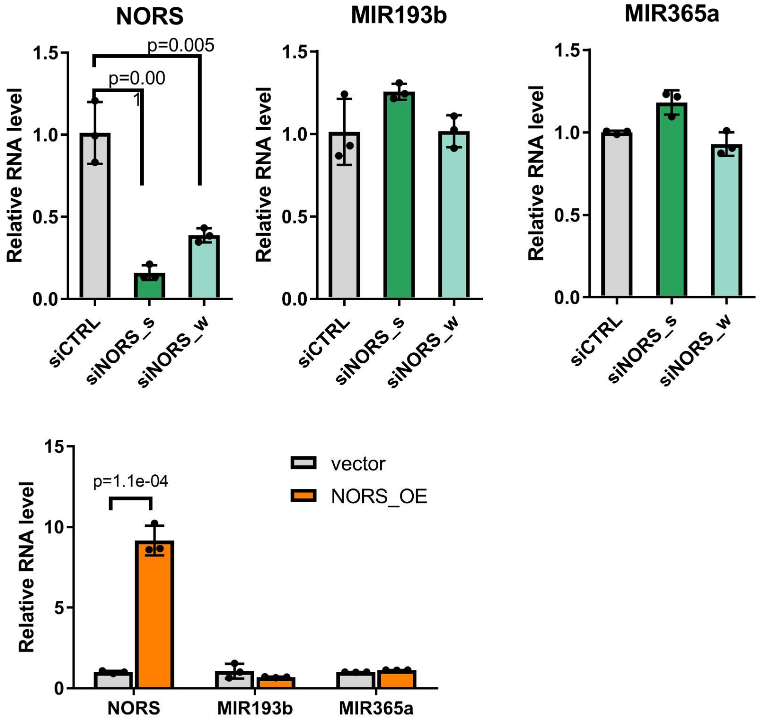

Supplementary Figure 7. *lincNORS* siRNAs target sequence and knockdown efficiency test.

(a) *lincNORS* cDNA and RNAi oligos sequence. Targeted regions of two siRNAs and one shRNA used to knockdown *lincNORS* were highlighted with indicated colors on *lincNORS* cDNA sequence.

(b) qPCR analysis of *lincNORS* intronic miR193b and miR365a level in MCF-7 cells with *lincNORS* knockdown or overexpression. Data represent mean ± SD from three biological replicates (Student's t-test).

Supplementary Figure 8

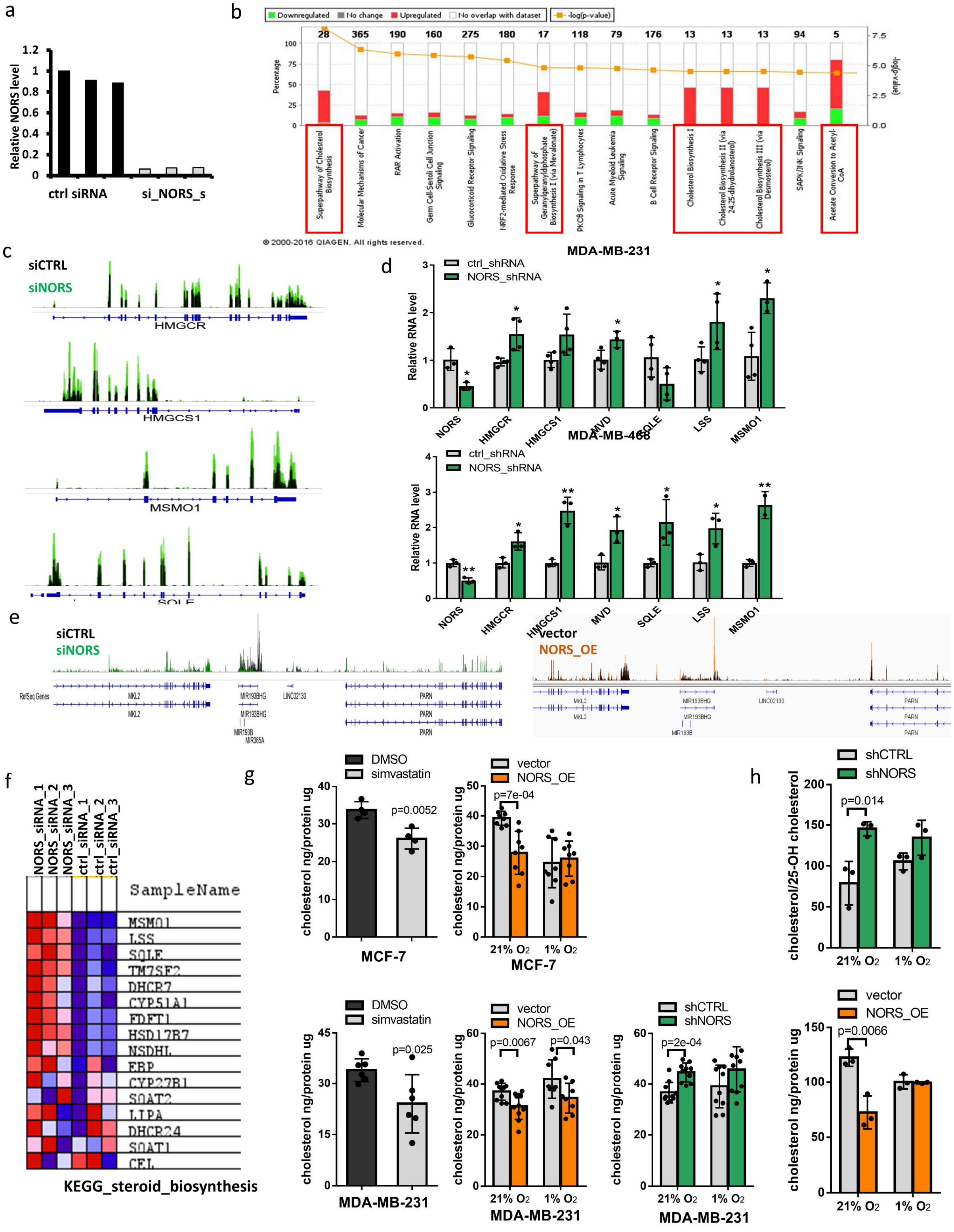

Supplementary Figure 8. Knockdown of MIR193BHG upregulated cholesterol/steroid biosynthesis genes.

(a) Knockdown of *lincNORS* by siRNA in the MCF-7 cells used in RNA-seq was confirmed by qPCR in each biological replicate.

(b) IPA analysis of differentially expressed genes in *lincNORS* knockdown MCF-7 versus control revealed by RNA-seq. Cholesterol synthesis-related pathways are marked with red rectangles.

(c) RNA-seq visualization showing expression of HMGCR, HMGCS1, MSMO1 and SQLE in MCF-7 cells transfected with *lincNORS* siRNA or control siRNA.

(d) qPCR analysis of selected cholesterol synthesis genes in MDA-MB-231 and MDA-MB-468 cells stably expressing *lincNORS* shRNA and 1% O2 for 24hrs. Data represent mean $\pm$ SD from three/four biological replicates (*p < 0.05, **p<0.01, two sided Student's t-test, exact P values are provided in the Source Data file).

(e) RNA-seq visualization showing expression of *lincNORS* and its neighboring genes in MCF-7 cells with *lincNORS* knockdown or overexpression.

(f) Heatmap depicting expression of genes in steroid biosynthesis (KEGG) in MCF-7 cells transfected with *lincNORS* siRNA or siRNA control.

(g) Total cholesterol in MCF-7 (top) or MDA-MB-231 (bottom) cells with *lincNORS* knockdown or overexpression was quantified using Wako Cholesterol assay and normalized to total protein content. Cells treated with simvastatin (1μM) or DMSO control were included as positive control for the assay. Data represent mean $\pm$ SD from 4~10 biological replicates (two-sided Student's t-test).

(h) LC-MS metabolite measurement of cholesterol/25-OH cholesterol ratio in MDA-MB-231 cells with stable *lincNORS* knockdown or overexpression. Data represent mean $\pm$ SD from three biological replicates (two-sided Student's t-test).

Supplementary Figure 9

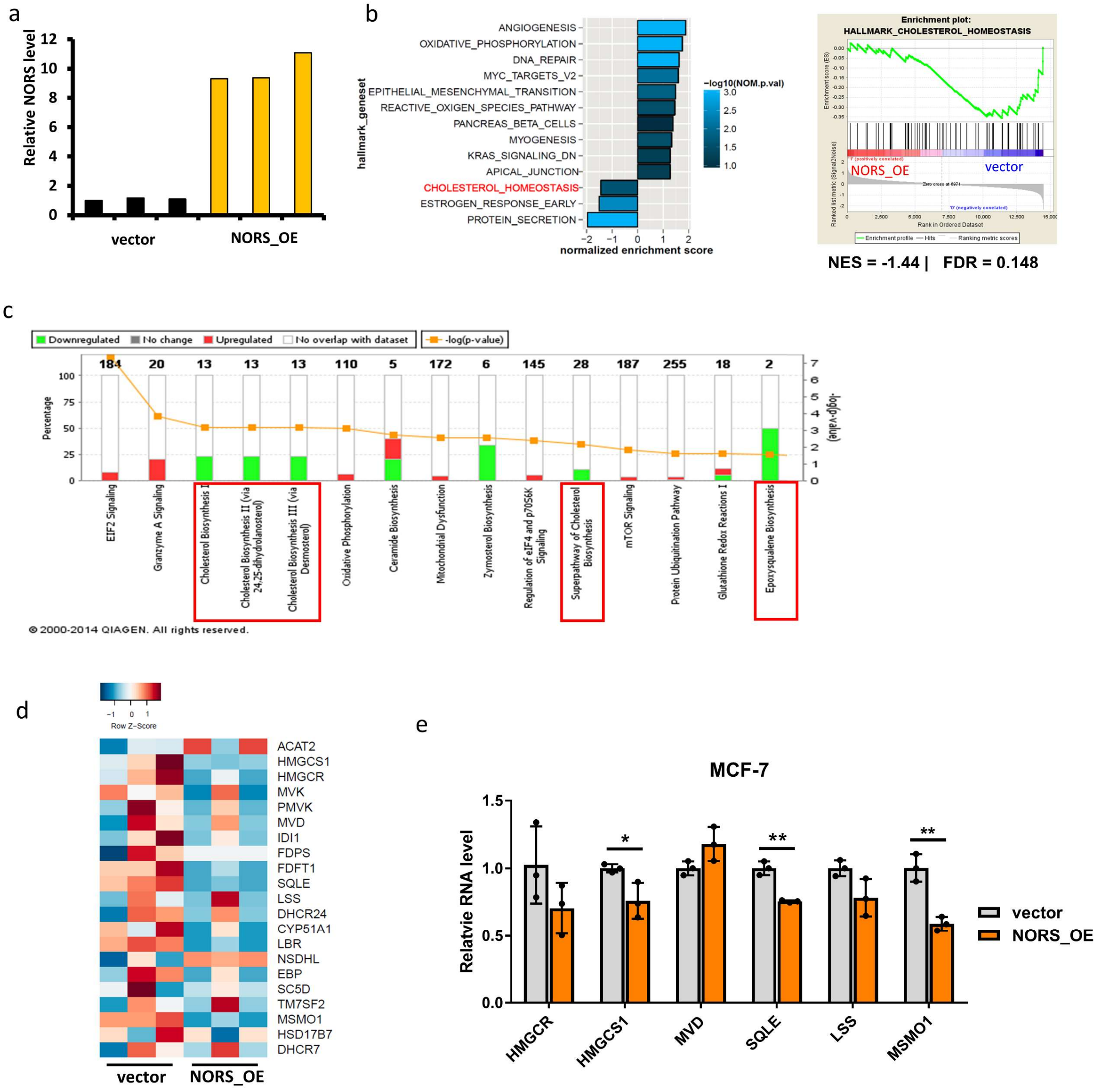

Supplementary Figure 9. *lincNORS* overexpression downregulated cholesterol synthesis pathway.

(a) Overexpression of *lincNORS* in the MCF-7 cells used in RNAseq was confirmed by qPCR in each biological replicate.

(b) Gene set enrichment analysis of hallmark gene sets associated with *lincNORS* overexpression versus the control in MCF-7. Enrichment plot of cholesterol homeostasis gene set is shown on the right.

(c) Heatmap showing expression of genes in cholesterol synthesis pathway in MCF-7 cells with *lincNORS* overexpression versus control vector.

(d) IPA analysis of differentially expressed genes in *lincNORS* overexpressing MCF-7 versus control revealed by RNA-seq. Cholesterol synthesis-related pathways are marked with red rectangles.

(e) qPCR analysis of selected cholesterol synthesis genes in MCF-7 cells with *lincNORS* overexpression. Data represent mean $\pm$ SD from three biological replicates (*p < 0.05, **p < 0.01, two-sided Student's t-test, exact P values are provided in the Source Data file).

Supplementary Figure 10

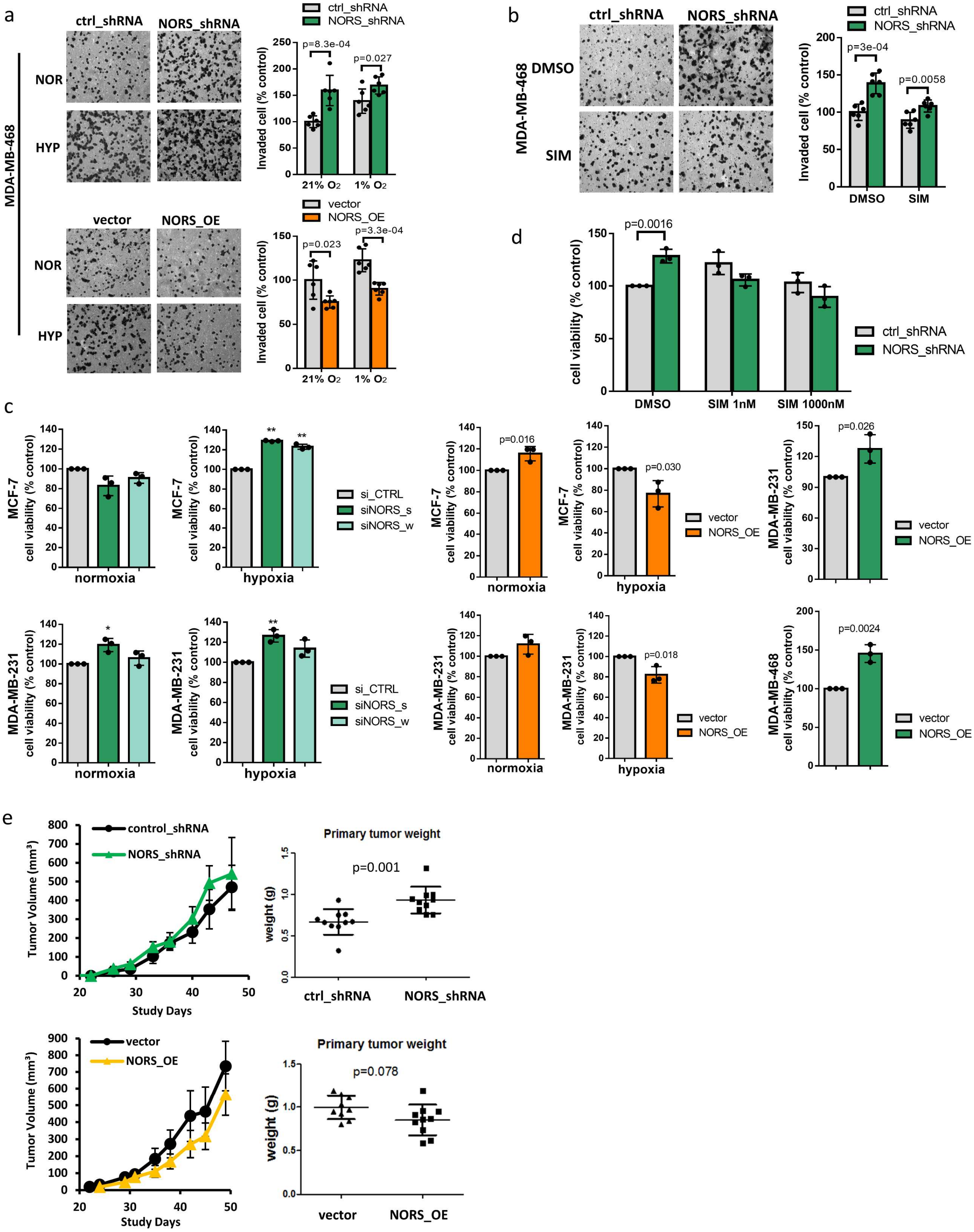

Supplementary Figure 10. *lincNORS* inhibits survival, invasion of breast cancer cells.

(a) MDA-MB-468 cells with either knockdown or overexpression of *lincNORS* were plated in Boyden chamber with Matrigel and cultured in 21% O2 or 1% O2. The cells that invaded through Matrigel were quantified 24hrs after plating. Data are shown as mean $\pm$ SD six biological replicates (two-sided Student's t-test).

(b) MDA-MB-468 cells with stable expression of *lincNORS* shRNA or control shRNA were plated in Boyden chamber with Matrigel with the presence of 1µM simvastatin or DMSO. The cells that invaded through Matrigel were quantified 20hrs after plating. Data are shown as mean $\pm$ SD six biological replicates (two-sided Student's t-test).

(c) *lincNORS* knockdown or overexpression cells were cultured in 21% O2 or 1% O2 for 48hrs. Cell viability was measured by MTT assay. Data are shown as mean $\pm$ SD from 3 independent experiments. For siRNA experiment one-way ANOVA with Dunnett's test was performed; for shRNA or overexpression experiments Student's t-test was performed (*p $<$ 0.05, **p$<$0.01).

(d) MDA-MB-468 cells with stable expression of *lincNORS* shRNA or control shRNA were cultured in 1% O2 for 48hrs in the presence of simvastatin or DMSO. Cell viability was measured by MTT assay. Data are shown as mean $\pm$ SD from three independent experiments (two-sided Student's t-test).

(e) Tumor growth curve and primary tumor weight in mice received either *lincNORS* knockdown (upper panel) or overexpressing (lower panel) MDA-MB-231. Data are shown as mean $\pm$ SD. N=10, two-sided Mann-Whitney U test.

Supplementary Figure 11

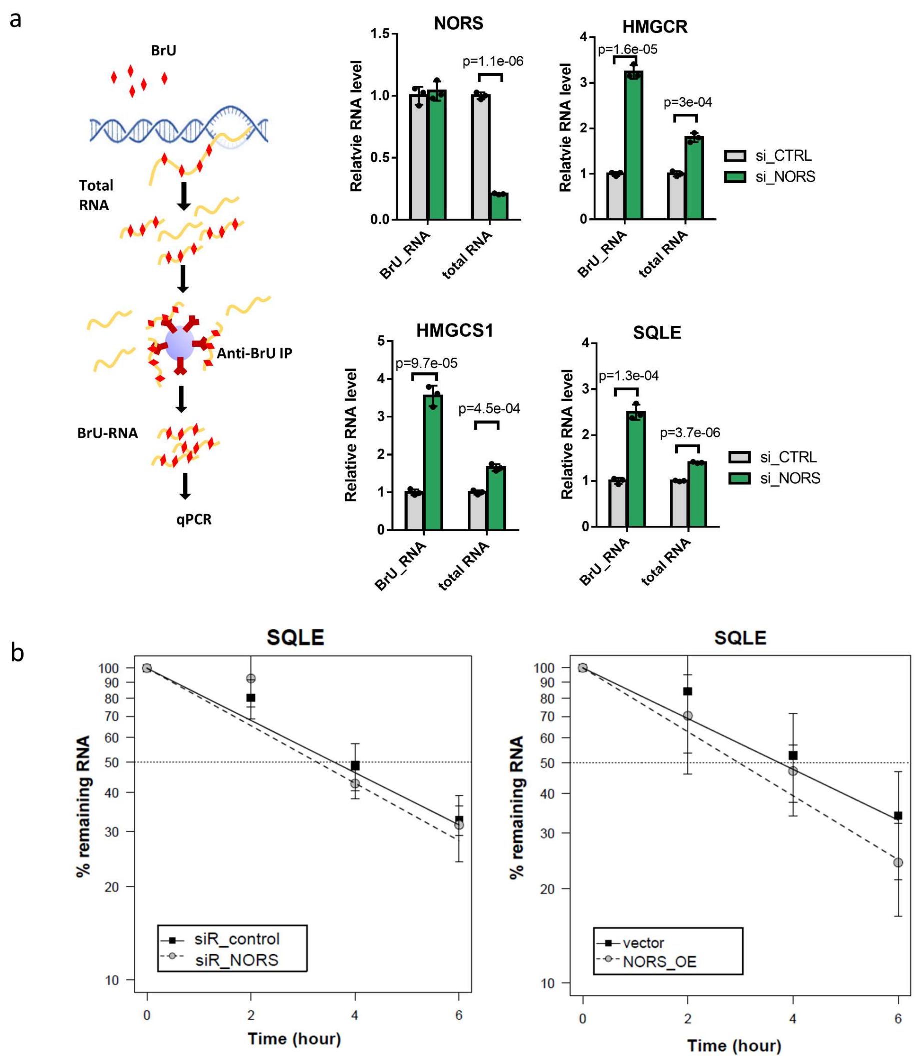

Supplementary Figure 11. *lincNORS* impacts cholesterol synthesis genes expression by suppressed transcription, not through RNA stability.

(a) The RNA level of HMGCR, HMGCS1, SQLE and *lincNORS* in newly synthesized RNA (BrU-RNA) and total RNA from hypoxic MCF-7 cells transfected with *lincNORS* siRNA or control siRNA was analyzed by qPCR. Data represent mean ± SD from three biological replicates (two-sided Student's t-test). Diagram illustrating the main steps in BrU-RNA Immunoprecipitation is shown on the left.

(b) SQLE RNA stability was measured as RNA degradation in the presence of 2.5µg/ml actinomycin D in MCF-7 cells with *lincNORS* overexpression or knockdown. Data represent mean ± SD from three biological replicates.

Supplementary Figure 12

a

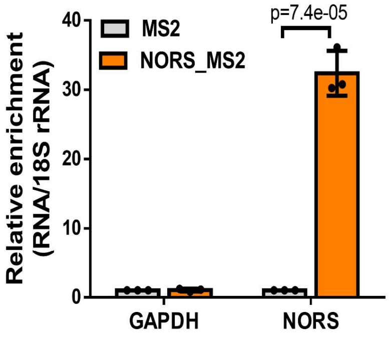

b

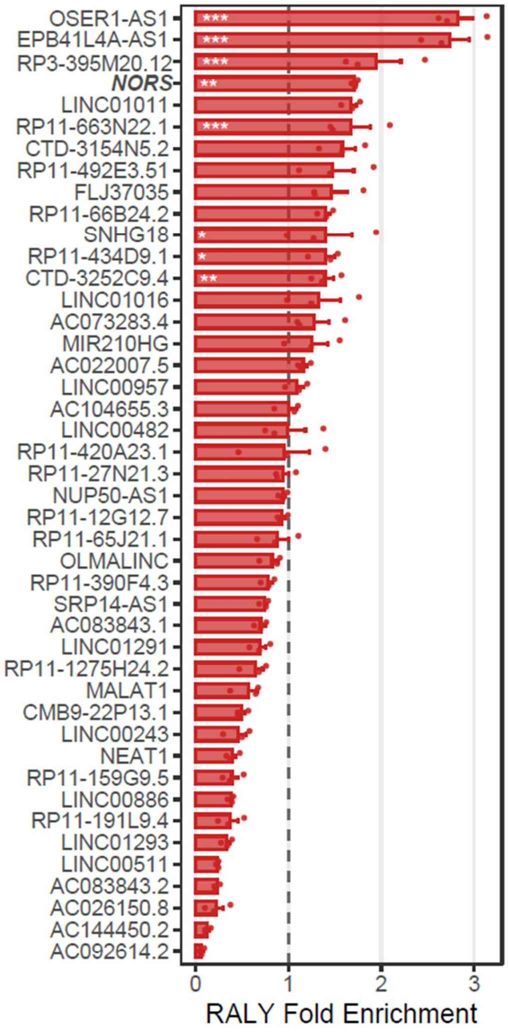

Supplementary Figure 12. *lincNORS* effect on cholesterol gene RNA synthesis was not through SREBP2 nor RNA stability regulation.

(a) The relative enrichment of *lincNORS* RNA in the MS2-pulldown complex was confirmed by qPCR. GAPDH RNA was included as negative control. Data represent mean ± SD from three biological replicates (two-sided Student's t test).

(b) Enrichment of 47 hypoxia-induced lincRNAs in RALY-containing immunoprecipitated complex. The barplot displays the mean fold enrichment ± S.E.M. from three independent RALY RIP-Seq vs control experiments in MCF7 cells. The statistical significance of the enrichment was determined with CuffDiff v2.2.1 (*FDR < 0.05, **FDR < 0.01, ***FDR < 0.001).

Supplementary Figure 13

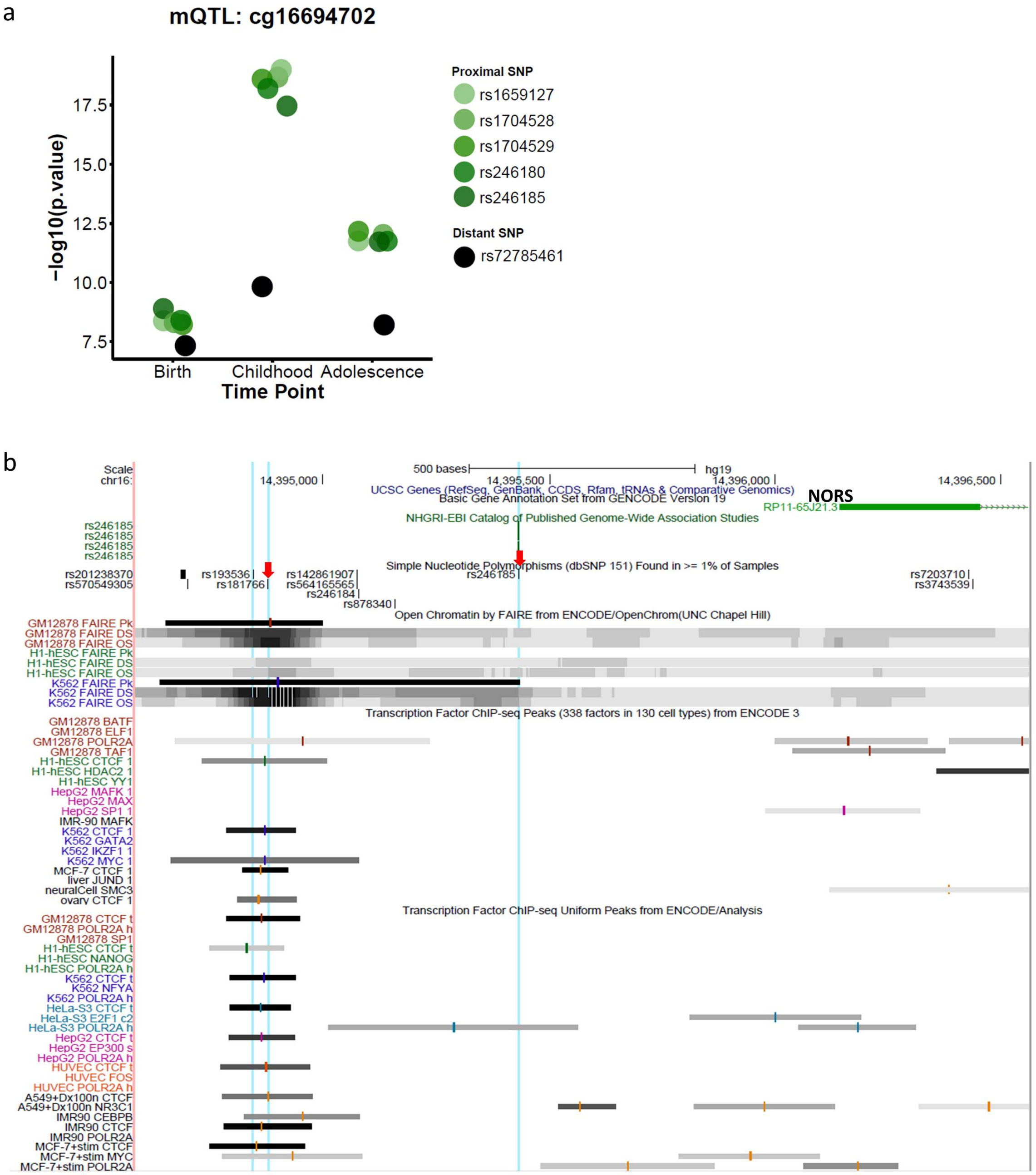

Supplementary Figure 13. Additional data showing connections between *lincNORS* and nearby SNPs.

(a) The association between the methylation status of cg16694702 and *lincNORS* proximal or distant SNPs across three time points in life. Data were downloaded from ARIES mQTL database (http://www.mqtldb.org/).

(b) UCSC genome browser view of overlaps between open chromatin, transcription factor binding sites with rs181766 and rs246185.
