# Supplementary material for: Regulation of cellular sterol homeostasis by the oxygen responsive noncoding RNA lincNORS
Source: Nat Commun. 2020 Sep 21;11:4755. doi: 10.1038/s41467-020-18411-x (PMC7505984; doi:10.1038/s41467-020-18411-x)
Supplement: Supplementary file 3 — Description of Additional Supplementary Files [file 41467_2020_18411_MOESM3_ESM.pdf]

Description of Additional Supplementary Files

File Name: Supplementary Data 1

Description: 47 hypoxia-regulated lincRNAs in MCF-7 (log2 FC >=1 or <=-1)

File Name: Supplementary Data 2

Description: NORS correlation with protein coding genes in tumor (TCGA)

File Name: Supplementary Data 3

Description: Bru-seq: MCF-7 Normoxia vs Hypoxia

File Name: Supplementary Data 4

Description: siNORS MCF-7 1% O2 RNAseq

File Name: Supplementary Data 5

Description: siNORS MDA-MB-468 1% O2 AmpliSeq

File Name: Supplementary Data 6

Description: NORS overexpression MCF-7 21% O2 RNAseq

File Name: Supplementary Data 7

Description: NORS-MS2 pulldown mass-spectrometry analysis

File Name: Supplementary Data 8

Description: Colocalization analysis: Evidence that the same causal SNP alters NORS expression and age of menarche

File Name: Supplementary Data 9

Description: Oligos information

File Name: Supplementary Data 10

Description: Antibody information
