# Supplementary material for: Regulation of cellular sterol homeostasis by the oxygen responsive noncoding RNA lincNORS
Source: Nat Commun. 2020 Sep 21;11:4755. doi: 10.1038/s41467-020-18411-x (PMC7505984; doi:10.1038/s41467-020-18411-x)
Supplement: Supplementary file 14 — Reporting Summary [file 41467_2020_18411_MOESM14_ESM.pdf]

nature research

Corresponding author(s): Mircea Ivan

Last updated by author(s): Jul 14, 2020

Reporting Summary

Nature Research wishes to improve the reproducibility of the work that we publish. This form provides structure for consistency and transparency in reporting. For further information on Nature Research policies, see our Editorial Policies and the Editorial Policy Checklist.

Statistics

For all statistical analyses, confirm that the following items are present in the figure legend, table legend, main text, or Methods section.

n/a	Confirmed	
☐	☒	The exact sample size (n) for each experimental group/condition, given as a discrete number and unit of measurement
☐	☒	A statement on whether measurements were taken from distinct samples or whether the same sample was measured repeatedly
☐	☒	The statistical test(s) used AND whether they are one- or two-sided<br>*Only common tests should be described solely by name; describe more complex techniques in the Methods section.*
☐	☒	A description of all covariates tested
☐	☒	A description of any assumptions or corrections, such as tests of normality and adjustment for multiple comparisons
☐	☒	A full description of the statistical parameters including central tendency (e.g. means) or other basic estimates (e.g. regression coefficient) AND variation (e.g. standard deviation) or associated estimates of uncertainty (e.g. confidence intervals)
☐	☒	For null hypothesis testing, the test statistic (e.g. F, t, r) with confidence intervals, effect sizes, degrees of freedom and P value noted<br>*Give P values as exact values whenever suitable.*
☒	☐	For Bayesian analysis, information on the choice of priors and Markov chain Monte Carlo settings
☒	☐	For hierarchical and complex designs, identification of the appropriate level for tests and full reporting of outcomes
☐	☒	Estimates of effect sizes (e.g. Cohen's d, Pearson's r), indicating how they were calculated

Our web collection on statistics for biologists contains articles on many of the points above.

Software and code

Policy information about availability of computer code

Data collection	Applied Biosystems 7900HT Real-Time PCR system software; Bio-Rad CFX Manager; Bio-Rad ChemiDoc imaging system; Illumina NextSeq500, Illumina HiSeq2000 sequencing platform.
Data analysis	HIF ChIP-Seq data was aligned to hg19 using Bowtie v1.2.1.1. RNAseq measuring hypoxia versus normoxia gene expression was aligned with TopHat v2.1.1; read counts was generated with HTSeq v0.6.1; differential gene expression analysis was performed with DESeq2 v1.12.3 in R (3.3.1). RNAseq data from samples with manipulated lincNORS was aligned with STAR V2.3.0 and differential expression was analyzed with EdgeR v3.8.6 in R (3.0.1). AmpliSeq data was generated using standard workflow of Torrent Suite Version 4.4 with AmpliSeq Transcriptome Plugin; differential expression analysis was performed with DESeq2 v1.12.3 in R(3.3.1).<br>IPA (QIAGEN) and GSEA (3.0) were used for pathway enrichment analysis.<br>RIP-Seq data were analyzed with CuffDiff v2.2.1 software.<br>Mass spectrometer data were analyzed with Proteome Discover 1.4 with SEQEST HT.<br>LC/MS data were analyzed with Agilent Masshunter Quantitative analysis software (V.B.06).<br>Tumor burden in mice lung and liver was analyzed with Aperio ImageScope_v11.2.0.<br>SNP colocalization analysis was performed in R(v3.4.2) with coloc package.<br>GraphPad Prism 6, Excel 2016, and R (3.3.1) were used to perform statistical analyses.

For manuscripts utilizing custom algorithms or software that are central to the research but not yet described in published literature, software must be made available to editors and reviewers. We strongly encourage code deposition in a community repository (e.g. GitHub). See the Nature Research guidelines for submitting code & software for further information.

Data

Policy information about availability of data

All manuscripts must include a data availability statement. This statement should provide the following information, where applicable:

- Accession codes, unique identifiers, or web links for publicly available datasets
- A list of figures that have associated raw data
- A description of any restrictions on data availability

Raw data of RNAseq and BrUseq data are deposited in the GEO database under the accession numbers GSE153293 and GSE152799, respectively. AmpliSeq data are provided as supplementary table s5. HIF ChIP-seq data are available from GSE28352. MCF-7 ChIA-PET data are available from GSE33664. RALY RIPseq data are available under Rossi et al. (2017) at DOI: 10.1093/nar/gkx235. All the data are available within the article or the supplementary information. The source data underlying the figures are provided either in supplementary table or in Source Data file. . All other relevant data are available from the corresponding author upon reasonable request.

Field-specific reporting

Please select the one below that is the best fit for your research. If you are not sure, read the appropriate sections before making your selection.

☒ Life sciences ☐ Behavioural & social sciences ☐ Ecological, evolutionary & environmental sciences

For a reference copy of the document with all sections, see nature.com/documents/nr-reporting-summary-flat.pdf

Life sciences study design

All studies must disclose on these points even when the disclosure is negative.

Sample size	The sample size was equal or larger that 3 in all cases. No method was used to calculate the sample size a priori. Sample size was chosen based on previous experience and standards in the field. We combined a number of analytical methods and standard statistical methods and difference was considered statistically significant when p-value was less than 0.05.
Data exclusions	No data were excluded from the analysis.
Replication	Each assay was generally done 3 times, unless stated otherwise. Transcriptomic analyses were done with different RNA-seq platforms and different cell types.<br>We used established signatures rather than a limited set of targets and multiple inactivation approaches of siRNA and shRNA that target non-overlapping regions of the lincNORS. This avoids pitfalls resulting from target overlaps.<br>The responses of lincNORS to hypoxia and hormones were independently verified by collaborators in different locations.<br>We verified that the GWAS SNPs in the regulatory region of the locus are highly reproducible for the hormonal phenotypes.<br>All attempts at replication were successful.
Randomization	For in vitro studies, cells for different groups were treated randomized. For in vivo study, animals with xenograft tumors were assigned randomly to experimental groups.
Blinding	Animal xenografts and pathological assessment were performed in a blinded manner by experts who were not involved in study design. For the other experiments, the investigators were not blinded to the experimental conditions since there are no subjective measures for the analyses.

Reporting for specific materials, systems and methods

We require information from authors about some types of materials, experimental systems and methods used in many studies. Here, indicate whether each material, system or method listed is relevant to your study. If you are not sure if a list item applies to your research, read the appropriate section before selecting a response.

Materials & experimental systems

n/a	Involved in the study	
☐	☒	Antibodies
☐	☒	Eukaryotic cell lines
☒	☐	Palaeontology and archaeology
☐	☒	Animals and other organisms
☒	☐	Human research participants
☒	☐	Clinical data
☒	☐	Dual use research of concern

Methods

n/a	Involved in the study	
☒	☐	ChIP-seq
☒	☐	Flow cytometry
☒	☐	MRI-based neuroimaging

Antibodies

Antibodies used	1. b-tubulin (Santa Cruz sc-5274, 1/1000): fig 1f, fig 6b<br>2 lamin B1 (ab16048, 1/1000): fig 1f, fig 2b, fig s4b, fig6b<br>3 acetyl-Histone H3 (Millipore 06-599, 1/1000): fig 1f, fig 6b<br>4. HIF-1a (R&D AF1935, 1/500): fig s4b<br>5 HIF-2a (R&D AF2886, 1/500): fig 2b; (Cell Signaling #7096, 1/500): fig s4d<br>6 RALY (ab170105, 1/1000): fig 6<br>7 TRIM28 (ab10483, 1/1000): fig 6a<br>8 SREBP2 (R&D AF7119, 1/1000): fig 6a<br>9 HnRNPC (Santa Cruz sc-32308, 1/1000): fig 6a<br>10 MS2 binding protein (Millipore ABE76, 1/1000): fig 6a<br>11 b-actin (Thermo MA5-15739, 1/1000): fig 6d; (Santa Cruz sc-1615, 1/2000): fig s4d<br>12 BrdU BD Pharmingen, 555627 BrUseq NA
Validation	Validations are based on the datasheets from the manufacturers.

Eukaryotic cell lines

Policy information about cell lines

Cell line source(s)	Human cancer cell lines MCF-7, MDA-MB-231, MDA-MB-468, T47D, A549, HT-29, SKOV3, 399 MIA-PaCa2, U118, and 293T cells were purchased from American Type Culture Collection (ATCC).<br>Patient-derived pancreatic cancer cell lines Pa03C, Panc10.05, Panc198, as well as cancer associated fibroblast cell line CAF19, were obtained from Dr. Anirban Maitra at Johns Hopkins University.<br>786-O cells were obtained from Dr. Maria Czyzyk-Krzeska (University of Cincinnati).<br>Human lung fibroblast cell line WI-38 was obtained from Dr. Shadia Jalal's lab (Indiana University School of Medicine).<br>Fallopian tube FTE282 epithelial cells (a gift from Dr. Ronny Drapkin, University of Pennsylvania).
Authentication	None of the cell lines used were autheticated.
Mycoplasma contamination	All cell lines were routinely tested for mycoplasma contamination (MycoAlert™ Mycoplasma Detection Kit, Lonza) and the results were negative each time.
Commonly misidentified lines<br>(See ICLAC register)	none

Animals and other organisms

Policy information about studies involving animals; ARRIVE guidelines recommended for reporting animal research

Laboratory animals	4-6 weeks old female NSG mice were used in this study. Mice were housed and bred in the animal facility under specific pathogen free conditions in a controlled environment of 12/12 hours light/dark cycle, 21 degrees Celsius, 55%–60% humidity, with chow and water ad libitum.
Wild animals	No wild animals were involved in this study.
Field-collected samples	No field-collected samples were involved in this study.
Ethics oversight	All animal experiments were approved and carried out in accordance with the Institutional Animal Care and Utilization Committee at Indiana University School of Medicine.

Note that full information on the approval of the study protocol must also be provided in the manuscript.
